# Interface-induced magnetic polar metal phase in complex oxides

**DOI:** 10.1038/s41467-019-13270-7

**Published:** 2019-11-20

**Authors:** Meng Meng, Zhen Wang, Aafreen Fathima, Saurabh Ghosh, Mohammad Saghayezhian, Joel Taylor, Rongying Jin, Yimei Zhu, Sokrates T. Pantelides, Jiandi Zhang, E. W. Plummer, Hangwen Guo

**Affiliations:** 1 Department of Physics & Astronomy, Louisiana State University, Baton Rouge, LA 70803 USA; 2 Condensed Matter Physics & Materials Science Department, Brookhaven National Laboratory, Upton, NY 11973 USA; 3 Department of Physics & Nanotechnology and SRM Research Institute, SRM Institute of Science and Technology, Kattankulathur, 603203 Tamil Nadu India; 4 Department of Physics and Astronomy and Department of Electrical Engineering and Computer Science, Vanderbilt University, Nashville, Tennessee 37235 USA; 5 Materials Science and Technology Division, Oak Ridge National Laboratory, Oak Ridge, Tennessee 37831 USA

**Keywords:** Electronic properties and materials, Electronic properties and materials, Magnetic properties and materials, Magnetic properties and materials, Surfaces, interfaces and thin films

## Abstract

Polar metals are commonly defined as metals with polar structural distortions. Strict symmetry restrictions make them an extremely rare breed as the structural constraints favor insulating over metallic phase. Moreover, no polar metals are known to be magnetic. Here we report on the realization of a magnetic polar metal phase in a BaTiO_3_/SrRuO_3_/BaTiO_3_ heterostructure. Electron microscopy reveals polar lattice distortions in three-unit-cells thick SrRuO_3_ between BaTiO_3_ layers. Electrical transport and magnetization measurements reveal that this heterostructure possesses a metallic phase with high conductivity and ferromagnetic ordering with high saturation moment. The high conductivity in the SrRuO_3_ layer can be attributed to the effect of electrostatic carrier accumulation induced by the BaTiO_3_ layers. Density-functional-theory calculations provide insights into the origin of the observed properties of the thin SrRuO_3_ film. The present results pave a way to design materials with desired functionalities at oxide interfaces.

## Abstract (web summary)

Polar metals—metals with polar structural distortions—are known not to be magnetic. Here, the authors demonstrate a magnetic polar metal phase in a BaTiO_3_/SrRuO_3_/BaTiO_3_ heterostructure displaying high conductivity and ferromagnetic ordering with high saturation moment.

## Introduction

In condensed matter physics, the itinerant electrons in metals lead to effective screening of local electric dipole moments, disfavoring the formation of long-range ordered dipoles, i.e. polar structural distortions^1–3^. Consequently, polar metals are rare in nature^3–6^. Inspired by the original work of Anderson and Blount^4^, an extensive search of polar metals has been conducted, especially in transition-metal compounds. In transition-metal oxides (TMOs), polar distortions exist in ferroelectric materials, which are typically insulating with empty d orbitals (e.g., d^0^ configuration)^7^. Metallicity can be induced via carrier doping by creating oxygen vacancies or replacing transition-metal ions. However, the amplitudes of polar distortions are suppressed rapidly, as has been shown in the cases of BaTiO_3−δ_ and PbTi_1−*x*_Nb_*x*_O_3_^8–10^. An alternative approach to achieve a stable polar metal phase is to induce polar distortions and metallicity from different types of ions. For example, in LiOsO_3_ bulk crystals^11^, polar distortions are induced at the A-site Li ions while metallicity is maintained with the partially filled B-site Os orbitals^1,2^. Similarly in NdNiO_3_(111) thin films, Nd off-centering and partially filled Ni orbitals are responsible for polar distortions and conductivity, respectively^12^. In both cases, the coupling strength between polar distortions and itinerant electrons is intrinsically weak as the two features originate from different ion sublattices^12^. However, LiOsO_3_ is highly toxic and unsuited for thin-film fabrication due to the high Li ionic mobility, while the conductivity of NdNiO_3_ (111) thin films is low. Notably, no polar metals in the literature possess any pronounced magnetism. This fact fits in the scenario that magnetism usually requires exchange interactions between partially filled d states *(*d^*n*^ configuration) where polar distortions are inhibited^13^.

In this article, we present an approach to establish a ferromagnetic polar metal phase using oxide heterostructures. Due to the strong interplay among spin, lattice, and charge degrees of freedom at the heterointerface^14–18^, in BaTiO_3_/SrRuO_3_/BaTiO_3_ (BTO/SRO/BTO) heterostructures, we demonstrate that polar distortions can be induced in 3-u.c. of SRO by interfacial effects as revealed by aberration corrected electron microscopy and spectroscopy. This 3-u.c. SRO exhibits high conductivity and ferromagnetism with high magnetic moment, thus forming a unique magnetic polar metal phase. Density-functional-theory (DFT) calculations provide insights into the origin of the observed properties of the thin SRO film. The polar distortions, metallicity, and magnetism in our system all originate from the Ru–O atomic layers so that intrinsic coupling among these features is expected. The present results not only can lead to ferroelectric-like functionality in a metallic system^19^, but also indicate a pathway to design materials with emergent functionalities^20,21^.

## Results

### STEM structural characterization

**Fig. 1:**
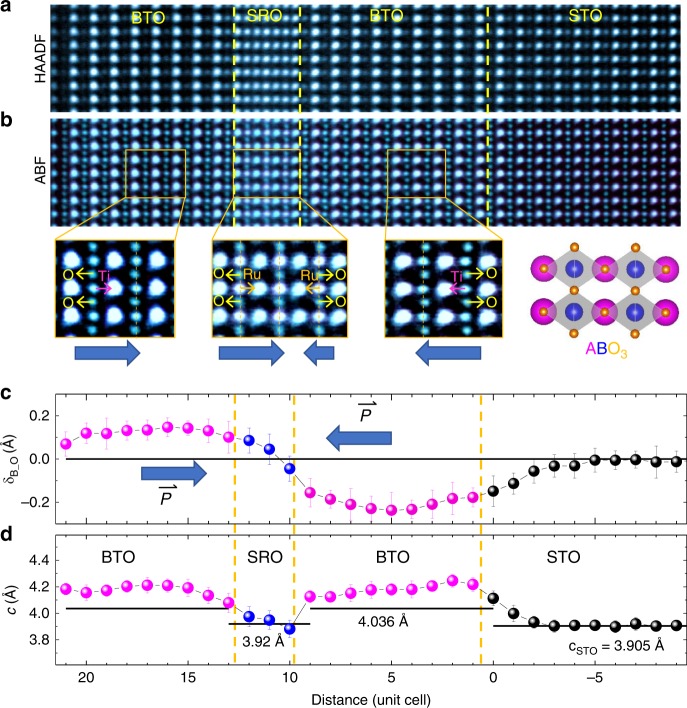
Direct visualization of interfacial polar distortions in the BTO 9/3/10 film. **a** HAADF- and **b** inversed ABF-STEM images taken along [110] direction. Enlarged ABF-STEM images showing polar distortions in the BTO and SRO blocks. Blue arrows indicate polar displacement direction. **c** Variation of polar displacements *δ*_B-O_ across the whole film. The polar displacements, *δ*_B-O_, refer to relative displacements between B site cations and O anions. **d** Out-of-plane lattice parameters, *c*, as a function of distance. The measurements were determined by averaging around 60 unit cells parallel to the interface. The error bar shows the standard deviations of the averaged measurements for each vertical atomic layer

A heterostructure consisting of 9 u.c. BTO/ 3 u.c. SRO/ 10 u.c. BTO (referred to as BTO 9/3/10 hereafter) is grown on a SrTiO_3_ (STO) substrate by pulsed laser deposition (see Methods). We first address lattice distortions in the heterostructure. Cross-sectional aberration corrected scanning transmission electron microscopy (STEM) combined with electron energy loss spectroscopy (EELS) serves as one of the most powerful tools to identify lattice distortions in ultrathin films across interfaces^22,23^. Figure 1a displays a high-angle annular dark field (HAADF)-STEM image of the BTO 9/3/10 film grown on a TiO_2_-terminated STO substrate. The image intensity is roughly proportional to the total atomic number *Z*^2^ in the column. The SRO block starts and terminates with a SrO layer in agreement with the well-known SRO growth mode^24^. Detailed EELS analysis illustrates that a small amount of chemical intermixing is mostly confined within one unit cell at both sides of the interfaces (Supplementary Note 1 and Supplementary Fig. 1).

Bulk BTO exhibits a polar structure where titanium and oxygen ions displace against one another, whereas bulk SRO has a nonpolar antiferrodistrotive (AFD) structure with tilted oxygen octahedra. To examine the structural evolution across the interfaces, we performed atomically resolved annular bright-field (ABF) STEM imaging which allows direct observation of the RuO_6_/TiO_6_ octahedral distortions across the whole film. As shown in Fig. 1b, the BTO layers show bulk-like Ti–O polar displacements reflecting their polar nature. Remarkably, the RuO_6_ octahedron tilts that exist in bulk are totally suppressed in the SRO layers. Instead, polar displacements are observed as induced by the BTO layers. Figure 1c displays the evolution of the layer-by-layer Ru–O/Ti–O polar displacements (*δ*_B–O_) across the whole film. The Ti–O polar displacements near SRO are about 0.2 ± 0.02 Å, and the Ru–O polar displacements are smaller, with a maximum value of 0.08 ± 0.04 Å. Moreover, convergent beam electron diffraction (CBED) in STEM mode was also performed to study the lattice distortions across the film. The comparison of experimental and simulated CBED (Supplementary Note 2 and Supplementary Fig. 2) indicates that the BTO blocks exhibit polar displacements and the SRO blocks have polar displacements plus octahedron rotations about the *c*-axis, consistent with our observations in Fig. 1. The direction of polarization is opposite in the BTO layers that sandwich the SRO layer, as indicated by the blue arrows in Fig. 1c, a head-to-head polar configuration is observed^25^. From both STEM and second harmonic generation measurements (Supplementary Note 3), we illustrate that such head-to-head polar configuration is the dominant phase in our heterostructure.

In-plane lattice parameters of the bulk BTO and SRO are larger than STO substrate (*a*_STO_ = 3.905 Å, *a*_SRO_ ~ 3.925 Å, and *a*_BTO_ ~ 3.992 Å)^26,27^, indicating that the film is subjected to compressive strain. The in-plane lattice parameters of SRO and BTO films were measured to be 3.90 ± 0.02 Å (Supplementary Fig. 4), revealing that the film grows coherently on the STO substrate as confirmed by X-ray diffraction data (Supplementary Fig. 5). As shown in Fig. 1d, the out-of-plane lattice parameters were measured to be 4.19 ± 0.02 Å for BTO, and 3.95 ± 0.02 Å for SRO, larger than those of the bulk values (c_BTO_ ~ 4.036 Å, c_SRO_ ~ 3.926 Å) due to compressive strain. The elongation of out-of-plane lattice parameters should produce an enhanced polarization in BTO and suppress octahedral tilts in SRO^28,29^. On the other hand, the reduced in-plane lattice parameters of the SRO film favor enhanced octahedral rotation about the *c*-axis^28^. These results are in agreement with the structural feature observed in ABF-STEM images.

### Transport and magnetic properties

**Fig. 2:**
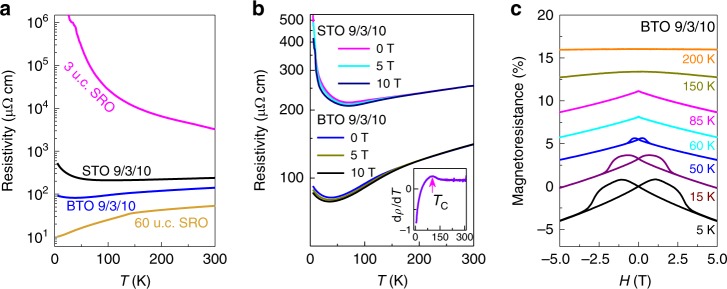
Transport properties in BTO 9/3/10 and STO 9/3/10. **a** Temperature dependence of longitudinal resistivity *ρ* for the BTO 9/3/10, STO 9/3/10, 3 u.c. SRO, and 60 u.c. SRO films. The BTO 9/3/10 has low resistivity compared to STO 9/3/10 and bare 3 u.c. SRO film. **b** Temperature dependence of longitudinal resistivity of BTO 9/3/10 and STO 9/3/10 under various magnetic field. Metallic behavior is observed at all fields. Inset: temperature dependence of the derivative d*ρ*/d*T* of BTO 9/3/10. A clear kink around 125 K is the typical feature of magnetic transition in SRO. **c** Magnetoresistance of BTO 9/3/10 at different temperatures. The butterfly loop indicates ferromagnetism in BTO 9/3/10

Bulk SRO is a good metal with low resistivity value in TMO families^30^. In epitaxial thin film, SRO becomes insulating below a critical thickness of 4–5 u.c.^31,32^. Figure 2a shows resistivity data of the BTO 9/3/10 sample as a function of temperature. A metallic behavior is clearly observed with resistivity in the order of ~100 μΩ cm in the measured temperature range. The small upturn in resistivity below 40 K is likely due to the localization effect of charge carriers as widely observed in ultrathin oxide films^33^. For comparison, a highly insulating behavior is observed in a bare 3-u.c. SRO film grown on a STO substrate. A thick (60 u.c.) SRO film on STO shows a metallic behavior with the resistivity in the order of 10 μΩ cm, comparable to bulk value^30^. Remarkably, as shown in Fig. 2a, the BTO 9/3/10 has a resistivity that is only within one order of magnitude larger than the bulk value. In contrast, the resistivity of STO 9/3/10 (9-u.c. SrTiO_3_/3-u.c. SrRuO_3_/10-u.c. SrTiO_3_ fabricated under the same conditions as BTO 9/3/10) is five times larger than that of BTO 9/3/10 at low temperatures and becomes insulating below 90 K.

We discuss the possible origins on the above observations. As discussed in Supplementary Note 5, both STO 9/3/10 and BTO 9/3/10 films have high quality with very low defect densities, ruling out different defect densities as the origin of observed phenomena. We also examined the capping effect by fabricating 3 u.c. SRO on STO substrate capped with 10 u.c. of BTO layers. As shown in Supplementary Fig. 7, the capping effect partially recovers the metallic behavior compared to bare SRO film, it is still more resistive than the BTO 9/3/10 samples. Therefore, the observed enhanced conductivity cannot be fully attributed to the capping effect. We measured the resistivity of three BTO 9/3/10 samples and similar transport behaviors are observed (Supplementary Fig. 8). To better understand the mechanism on the different transport behavior between BTO 9/3/10 and STO 9/3/10, we performed STEM characterization on the STO 9/3/10 sample. As shown in Supplementary Fig. 9, both the STO and SRO layers do not show any polar distortions, consistent with the nonpolar nature of STO. The observed conductivity enhancement in BTO 9/3/10 compared to STO 9/3/10 can be understood in the scenario of interface-induced carrier accumulation in the SRO layers by the electric field effects from ferroelectric BTO layers. The electric field from ferroelectric BTO can electrostatically modulate the carrier concentration in neighboring SRO layers, thus causing the conductivity increase in our system. Such electrostatic-induced conductivity enhancement has been observed by Ahn et al. in PZT/SRO system^34^. In Ahn et al.’s work, the enhancement of conductivity was observed when the polarization of PZT is pointing towards the SRO film, which is consistent with our polarization configurations at both BTO/SRO interfaces. In contrast, in STO 9/3/10, this effect is absent since no ferroelectric layers exist as shown in Supplementary Fig. 9. Therefore, it is plausible that ferroelectric electrostatic effect plays one of the most important roles in the improved metallicity in BTO 9/3/10.

Figure 2b shows the resistivity of BTO 9/3/10 and STO 9/3/10 under different magnetic fields applied perpendicular to the film surface. Compared to the zero-field case, the resistivity of BTO 9/3/10 under field has little difference at high temperatures, but is suppressed below *T*_C_ ~ 125 K which is defined as the onset of zero-field dρ/dT (inset), indicating that the magnetoresistance (MR) is negative below *T*_C_. Figure 2c displays the field dependence of MR at constant temperatures above and below *T*_C_. Note that negative MR depends almost linearly on *H* below *T*_C_, and shows a butterfly shape characteristic below 85 K. These features suggest that BTO 9/3/10 has ferromagnetic order below *T*_C_^35,36^.

**Fig. 3:**
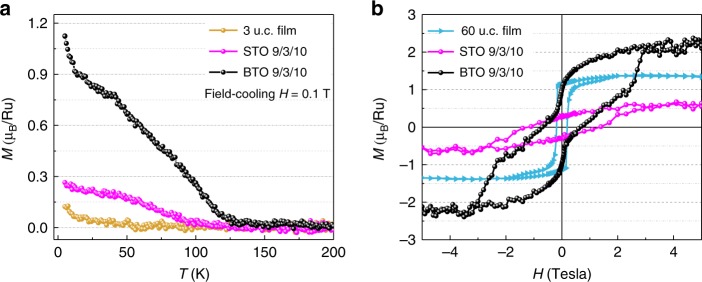
Unusual magnetism in BTO 9/3/10. **a** Temperature dependence of magnetization for BTO 9/3/10, STO 9/3/10 and a bare 3 u.c. SRO film under field-cooling (FC) measured by applying a magnetic field *H* = 0.1 T normal to the film plane. A pronounced magnetic transition at 125 K is shown in BTO 9/3/10. In contrast, STO 9/3/10 and bare 3 u.c. SRO show small magnetic moments. **b** Magnetic hysteresis loop measured at 5 K for the BTO 9/3/10 with saturated moment of ~2.3 μ_B_/Ru. The STO 9/3/10 shows weak ferromagnetism with saturated moment of ~0.6 μ_B_/Ru and the 60 u.c. SRO film shows normal ferromagnetism with saturated moment of ~1.4 μ_B_/Ru. Note that BTO and STO are diamagnetic in the entire temperature range investigated here

To confirm the magnetic nature of BTO 9/3/10, we measure the magnetization (*M*) as a function of temperature (*T*) and external magnetic field (*H*). While BTO is diamagnetic, SRO is known to be ferromagnetic in thick films below ~150 K, but becomes nonmagnetic below a critical film thickness of 4 u.c.^31,37^. As shown in Fig. 3a, the magnetic moment of a bare 3-u.c. SRO film grown on a STO substrate is small and has no sign for any magnetic transition. In contrast, *M*(*T*) for BTO 9/3/10 reveals a sharp rise below *T*_C_ ~ 125 K, indicating a paramagnetic-to-ferromagnetic phase transition. For comparison, the STO 9/3/10 shows a magnetic onset around 100 K with much smaller magnetic moments. To further confirm the ferromagnetic nature of the transition, we examine magnetization hysteresis for BTO 9/3/10, STO 9/3/10 and 60 u.c SRO film, as shown in Fig. 3b. At 5 K, the 60 u.c. SRO film shows typical bulk-like ferromagnetic behavior with a saturated magnetic moment of 1.4 μ_B_/Ru, corresponding to the low-spin Ru magnetic state as reported previously^30^. The STO 9/3/10 only shows a small hysteresis loop with saturated magnetic moment of 0.6 μ_B_/Ru, confirming its weak ferromagnetism and consistent with a previous publication^38^. Remarkably, the BTO 9/3/10 shows ferromagnetic hysteresis loop with a large saturated magnetic moment of 2.3 μ_B_/Ru at 5 T.

Two interesting features can be captured from the magnetization data. First, in BTO 9/3/10, the *M*(*H*) hysteresis loop is unusual, revealing a steep rise around 2 T (drop around −2 T) before reaching the saturation value of 2.3 μ_B_/Ru. The *M*(*H*) highly resembles the hysteresis loop of coupled soft/hard magnetic bilayer system^39^. In our SRO, the soft and hard magnetic components are likely originated from different amplitudes or direction of structure distortions in the middle SRO unit cell and two SRO unit cells near the interfaces (Fig. 1c). When the field is larger than 3 T, the Zeeman energy is large enough to align the spin in different layers, resulting in high saturation moment. Nonetheless, the high saturation moment has not been observed before in ultrathin SRO and is clearly beyond the saturation moment in bulk crystal or thick SRO films^30^. This feature indicates that BTO 9/3/10 is in favor of ferromagnetism in making the saturation moment close to the effective *S* = 1, which corresponds to the intermediate spin state of Ru^4+^. We also performed EELS measurements and determined that the valence of Ti remains close to 4+, further excluding the magnetic contribution from BTO layers (Supplementary Fig. 10). Second, in the weak ferromagnetic STO 9/3/10 sample, both STO layers are nonpolar so that no polar distortions are induced in the bracketing 3-u.c. SRO layer. Replacing the STO layers with BTO layers successfully induces not only polar distortions in the 3-u.c. SRO, but also strong ferromagnetism with high saturation moment.

## Discussion

Given the experimental observations that we can drive the SRO layer into a ferromagnetic polar metal configuration, it would be helpful to have a first-principles calculation to improve our understanding of the current system. In bulk SRO or thick SRO films, DFT-based calculations are capable of explaining observed properties without using a Hubbard-*U* parameter or by using a very small *U* of 0.6 eV^40–42^. In contrast, when the thickness of SRO is reduced to several unit cells, the theoretical calculations are inconclusive. Si et al. illustrated that DFT + *U* fails to explain the properties of ultrathin SRO film, requiring a DFT + DMFT calculation for better description^43^. Through the calculation, they conclude that ultrathin SRO is an antiferromagnetic insulator and no standard manipulation technique can restore ferromagnetism or metallicity except for carrier doping. Rondinelli et al. also found that *U* = 0.6 eV works well for bulk SRO, which is a metal, but they were unable to account for the observed metal-to-insulator transition in epitaxial films with thickness smaller than 4–5 u.c^42^. On the other hand, Gupta et al. pointed out that the metallicity of 3 u,c. SRO film can be retained by subjecting modest compressive strain with a DFT + *U* calculation with *U* = 2.5 eV which is a much larger value than bulk SRO^32^. The authors concluded that this metallic state is highly confined in two dimensions and spin polarized. Similar properties have been predicted using DFT + *U* calculation by Verissimo–Alves et al. on STO/SRO superlattices with *U* = 4.0 eV^44^. All the mentioned references showed that octahedral rotations are important in describing the electronic structure.

Given that our thin SRO layer exhibits a unique magnetic polar metal phase which has not been reported before, theoretical explorations regarding the origin of the observed phenomena would be valuable, even in the absence of a proven suitable functional for thin SRO films. Considering the special complexity of our heterostructure including the coupling between different possible distortion modes in SRO, we created a constrained model in an attempt to probe why a 3 u.c. SRO film sandwiched between BTO of opposite ferroelectric polarizations turns into a magnetic polar metal. As described in detail in the Supplementary Note 11 and 12, we have considered initial structures with possible modes guided by the symmetry and optimized the geometry within the LDA + *U* method. The three structural models in Supplementary Fig. 11b (i, ii, iii) are included in Supplementary Data 1, 2, and 3. The end BTO layers are not allowed to relax to ensure that opposite polar displacements are maintained. Finally, we relaxed the remaining structure with different *U* values. We found that the experimental results can be qualitatively reproduced with *U* = 3.5 eV (see Supplementary Figs. 11–16).

The approach presented here shows an emerging route to accelerate the discovery towards multifunctional materials with superior properties. Such approach can be easily transferable to a large expansion of other TMO materials such as Mn, Cu, Co, Ni oxides^23^. It is not only possible to induce a polar phase in ultrathin oxide compounds, but also to manipulate various types of electronic and magnetic interactions. For example, one of the immediate future developments is to switch the relative orientation of two BTO polarizations to control the structural, electronic, and magnetic properties of the bracketing SRO. Such development can open up the possibility to explore a branch of multiferroics by controlling magnetic and electronic properties in a metallic phase^19^. A recent publication shows active control of interfacial skyrmions in bilayer BTO/SRO, illustrating the importance of such interfaces^21^. We also anticipate this approach to bring further fruitful study to explore functionalities such as magnetoelectric resistive switching memories and ultrathin magnetism manipulation, etc.

In summary, we report a previously unknown magnetic polar metal phase in a complex-oxide heterostructure. The polar distorted SRO shows high conductivity compared to nonpolar SRO of the same thickness. Moreover, a ferromagnetic state with high saturation moment is observed. Polar distortions are found to be responsible for the emergent ferromagnetism, revealing the intrinsic structure–property relationship. The present findings show an emergent route towards designing materials with multiple functionalities.

## Methods

### Film growth, x-ray diffraction, and magnetic measurements

The BTO/SRO/BTO sandwich was grown on SrTiO_3_ (001) single crystal substrates using stoichiometric targets of BaTiO_3_ and SrRuO_3_ by pulsed laser deposition. The substrates were treated for 30 s in buffered hydrogen fluoride and annealed at 950 °C in 0.1 MPa oxygen atmosphere. During growth, the substrate temperature was held at 700 °C with laser energy density ~1 J cm^−2^. BTO was grown at 10 mTorr and SRO was grown at 80 mTorr, both mixed with 1% ozone. Reflection high-energy electron diffraction (RHEED) was used during growth to monitor oscillations and surface quality. After growth, samples were cooled down to room temperature at 100 mTorr oxygen/o-zone atmosphere. X-ray diffraction measurements have been performed on a laboratory double-axis diffractometer. Magnetic measurements are performed in Quantum Design Magnetic Property Measurement System (MPMS) from 10 to 300 K, by using reciprocating sample option (RSO) with the sensitivity of 5 × 10^−9^ emu.

### STEM image and EELS spectrum acquisition

Cross-sectional TEM samples were prepared using focused ion beam (FIB) with Ga + ions to a thickness around 40 nm and then further milled using Nanomill with low-energy Ar + ions to remove the surface damage on the samples. The STEM and EELS results presented in the paper were obtained using the 200 kV JEOL ARM electron microscope at Brookhaven National Library equipped with double aberration correctors, a dual-energy-loss spectrometer and a cold field emission source. The atomic-resolved STEM images were collected with a 21 mrad convergent angle (30 μm condenser aperture) and a collection angle of 67–375 mrad for HAADF and 11–23 mrad for ABF imaging. The STEM imaging conditions were optimized for simultaneous EELS acquisition with a probe size of 0.8 Å, a convergence semi-angle of 20 mrad, and the collection semi-angle of 88 mrad. EELS spectroscopy images were obtained across the whole film with a step size of 0.2 Å and a dwell time of 0.05 s per pixel. The position of each atom column in the HAADF- and ABF-STEM images is measured by the two-dimensioanl Gaussian function. The lattice parameters were extracted from A-site positions, and the polar displacements were calculated by oxygen position with respect to the B-site position. The fine structure of the EELS spectrum (Ti-L edge) was obtained with an energy resolution about 0.7 eV with energy dispersion of 0.25 eV. Dural EELS mode was used to calibrate the energy level of the core-loss peaks with the zero-loss peak and to detect possible chemical shifts in the films. Background of the EELS spectra were subtracted using a power-law function, and multiple scattering was removed by a Fourier deconvolution method.

### SHG measurement

SHG measurements were conducted in reflection geometry with an incident angle of 45° and fluence of 8 mJ/cm^2^. The experimental setup is shown in Fig. S3. After reflection, the fundamental field was filtered and the S- and P-polarized components of the generated second harmonic were individually isolated using a Glan–Taylor polarizer. Optical pulses are provided via commercially purchased Solstice Ace Ti:Sapphire amplifier system from Spectra-Physics, generating pulses at 1.55 eV with a width of <100 fs at a 1 kHz frequency. The second harmonic was detected using a high-sensitivity spectrometer with a charge-coupled device. For polarization anisotropy measurements, each sample crystalline angle was fixed so that the relative angle between the in-plane crystal axis and the fundamental field was identical. The fundamental field polarization was rotated using a high-accuracy rotation stage.

### Density-functional calculations and model

First-principles calculations were carried out using DFT^45^+*U* (d–d static Coulomb interaction)^46^ method with projector augmented wave (PAW) potentials^47^ using the PBE exchange-correlation functional^48^, as implemented in the Vienna ab initio simulation package (VASP)^49^. The total energy and Hellman–Feynman forces were converged to 1 μeV and 0.02 eV Å^−1^, respectively. All calculations were performed with a 450-eV energy cutoff and a Γ-centered 4 × 4 × 2 k-point mesh. We have considered $\sqrt 2 \;a\; \times \;\sqrt 2 \;b\; \times \;16c$ supercell of the pseudocubic unit cell. The system contains a total of 160 atoms. Here, fixed the *a* and *b* are the in-plane lattiec to the bulk SrTiO_3_ value (*a* = 3.905 Å). First, we have considered <P+/SrRuO_3_/P->configuration where the SRO region is kept in the cubic structure (i.e., without any distortion in the SRO block) and BTO in the bulk tetragonal structure (i.e. with the polar distortion). The calculated length of <P+/SrRuO_3_ (no distortion) /P-> configuration from experimentally determined building block *c*-axis lattice parameters (i.e., unit cell *c*-axis 4.19 Å for BTO, and 3.95 Å for SRO, the *c*-axis has been obtained for 6/3/7 structure as 13 (u.c) × 4.19 + 3 (u.c.) × 3.95 = 66.32 Å) is found to be 66.32 Å. We have optimized the length of simulation cell along the *c*-axis of <P+/SrRuO_3_ (no distortion) /P-> configuration keeping *a* and *b* fixed at STO in-plane lattice value (*a* = 3.905 Å) as shown in Supplementary Fig. 13. The DFT optimized length turns out to be 67.36 Å which is great agreement of (1.56% of overestimation) compared to experimental *c*-axis. We have kept the *c*-axis of the supercell to 67.36 Å all other calculations.

## Supplementary information

Supplementary Information

Description of Additional Supplementary Files

Supplementary Data 1

Supplementary Data 2

Supplementary Data 3

## Data Availability

The data and code that support the findings of this study are available from the corresponding authors upon reasonable request.
