# Supplementary material for: Interface-induced magnetic polar metal phase in complex oxides
Source: Nat Commun. 2019 Nov 20;10:5248. doi: 10.1038/s41467-019-13270-7 (PMC6868157; doi:10.1038/s41467-019-13270-7)
Supplement: Supplementary file 1 — Supplementary Information [file 41467_2019_13270_MOESM1_ESM.pdf]

Supplementary Information:

Interface-induced Magnetic Polar Metal Phase in Complex Oxides

Meng et al.

Supplementary Note 1: Elemental mapping of BTO 9/3/10 film

STEM/EELS elemental mapping was performed to study the chemical distribution across the film. Supplementary Figure 1 displays atomic-resolution elemental maps across the BTO 9/3/10 on STO substrate. As indicated by the yellow dotted line, the STO substrate terminates with a TiO_2 layer at the BTO/STO interface. One monolayer of Sr and Ba intermixing was observed in the STO side. The orange and red dotted lines indicate the bottom and top BTO/SRO interfaces, respectively. At the bottom (right-side) SRO/BTO interface, BTO terminates with a TiO_2 layer, and SRO starts with a SrO layer. This SrO/TiO_2 interface is very sharp and almost no chemical intermixing occurs. At the top (left-side) BTO/SRO interface, SRO also has a SrO termination layer, but with small amounts of Ba diffusion.

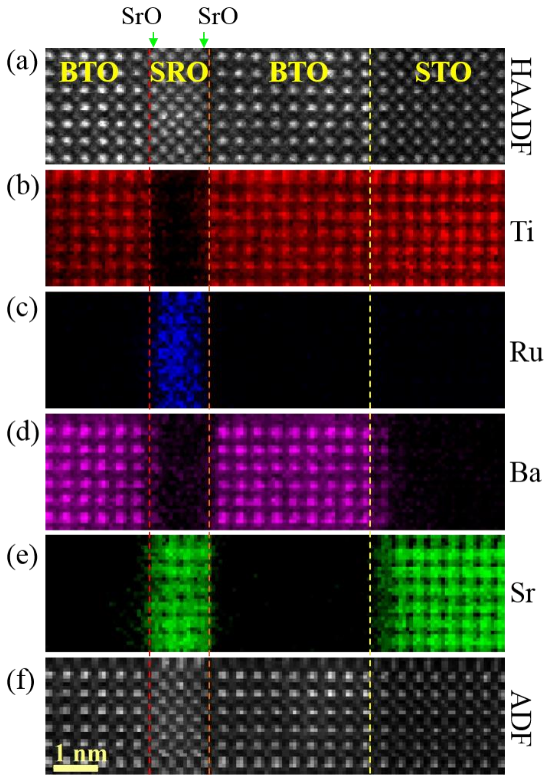

Supplementary Figure 1: STEM/EELS elemental maps across the BTO 9/3/10 on STO (001) substrate. **a** HAADF-STEM image taken along [100] direction. EELS elemental maps from **b** Ti $L_{2,3}$ edge, **c** Ru $M_{2,3}$ edge, **d** Ba $M_{2,3}$ edge and **e** Sr $L_{2,3}$ edge and **f** simultaneously obtained ADF image. The yellow dotted line marks the interface between STO substrate and the film. The left-side (top) TiO_2/SrO and right-side (bottom)SrO/TiO_2 interfaces are indicated by the orange and red dotted lines.

Supplementary Note 2: Structural distortion verified by convergent-beam electron diffraction (CBED) pattern of the film

We also carried out position averaged convergent beam electron diffraction (PACBED) in STEM to further analyze the structural distortions of the BTO 9/3/10. The group symmetry of the structure can be characterized by the CBED. In order to separate the diffraction discs in the CBED pattern, a convergence angle of 4.9 mrad was used in our experiment, leading to a spatial resolution about 1nm.

Supplementary Figure 2 (d-f) shows the simulated patterns of different structures: cubic STO without distortion; BTO with polar structure; and SRO with rotation $a^0a^0c^+$ and polar ($Q_{Rot} + Q_{Polar}$). From the simulated diffraction patterns, the intensity of 0kl (k + l = 2n + 1) diffraction discs, such as 010 and 001, can be used to distinguish the lattice distortions. It is shown that the 010/001 discs intensity is too weak to be seen in the STO; and the intensity is highest in the BTO due to the polar structure; the intensity in the SRO is relatively weaker, which is induced by small Q_{Polar} and Q_{Rot}. The 0-10, 001, 0-21, and 0-12 discs overlapped with the 0-11 disc, we can focus the fine features in the 0-11 discs, as indicated by the arrows. Fig. S2 (a-c) displays PACBED patterns from STO substrate, BTO blocks and SRO blocks in the film, respectively. Compared with the simulated pattern, the STO substrate show a cubic structure without distortion; the clear fine features within 0-11 disc in the BTO blocks reveal the polar structure. For the SRO blocks, the fine features are weaker, which is induced by combination of the smaller polar displacements and the rotation along c-axis. The structural features extracted from the experimental PACBED diffraction patterns are consistent with our STEM images and DFT calculation results.

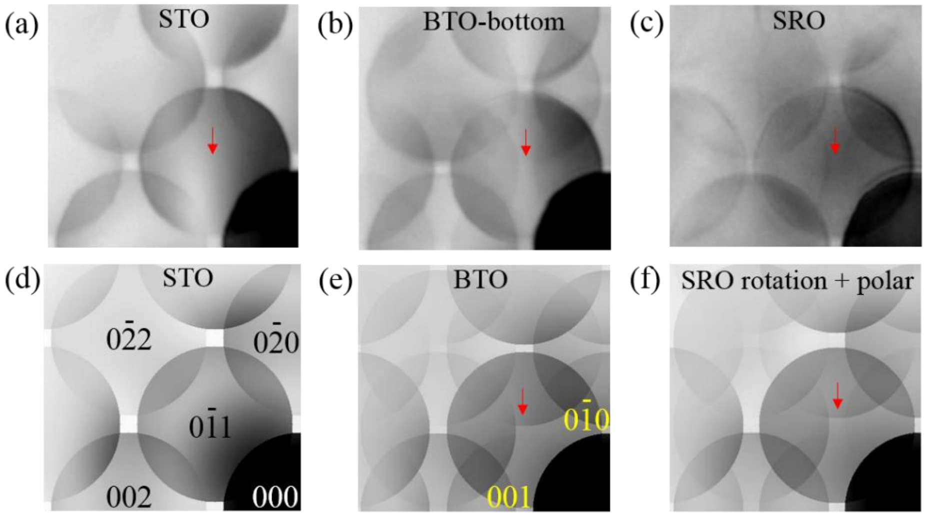

Supplementary Figure 2: Experimental PACBED patterns of the **a** STO substrate, **b** BTO blocks and **c** SRO blocks taken from the [100] direction. The acquisition step was 0.19 nm. To improve the signal to noise ratio, the patterns from each block are averaged. Simulated CBED patterns of **d** STO, **e** BTO with polar structure, and **f** SRO with polar and rotation structure. The diffraction discs are indexed in pseudo-cubic notation.

Supplementary Note 3: STEM and SHG determination on polar configuration

In Fig. 1, in order to make the STEM image clear to display the phenomena, we only showed a few unit cells of our heterostructure. In fact, we examined two TEM samples from (110) and (100) direction, both in the dimension of 7 μm in length and 30 nm in width. In both sample cuts, the existence of polar distortion and head-to-head configuration in both BTO layers are observed in the whole area. No other polar configurations or domain formations are observed. These results indicates that on the scale of micrometer level, the head-to-head polar distortion is the universal behavior of our BTO 9/3/10 film.

To understand the macroscopic polar configuration, we performed second harmonic polarimetry measurements. The experimental setup is shown in Supplementary Figure 3a. The generalized form for the second harmonic polarization $P(2\omega)$ is expressed by,

$$P(2\omega) \propto \sum_{i,j,k} F(2\omega)E_i(2\omega)\,\chi^{(2)}_{i,j,k}E_j(\omega)E_k(\omega)F(\omega)F(\omega). \quad (1)$$

Where χ is the second order susceptibility tensor; $E(\omega)$ and $E(2\omega)$ is the electric field for an 800 nm and 400nm photon, respectively. In Supplementary equation (1), the Fresnel coefficients $F(\omega)$ and $F(2\omega)$ are treated as a constant for constant fundamental field incident angle $\theta = 45^o$. The sample angle was held constant at an angle of $\beta = 18^o$. The fundamental field $E_i(\omega)$ and second harmonic fields $E_{i,p}(2\omega)$ and $E_{i,s}(2\omega)$ are [2],

$$E_i(\omega) = \begin{bmatrix} cos(\varphi)cos(\theta) \\ sin(\varphi) \\ cos(\varphi)sin(\theta) \end{bmatrix} and\ E_{i,p}(2\omega) = \begin{bmatrix} 1 \\ 0 \\ 1 \end{bmatrix}, E_{i,s}(2\omega) = \begin{bmatrix} 0 \\ 1 \\ 0 \end{bmatrix} \quad (2)$$

where φ is the angle of the polarization with $\varphi = 0$ defined as P polarization and $\varphi = 90$ defined as S polarization, as shown in Supplementary Figure 3a. For a *mm2* symmetry, the second order

susceptibility tensor reduces to five independent nonzero elements (taking into account the Kleinman symmetry approximation): $\chi_{xxz}^{(2)}$, $\chi_{yyz}^{(2)}$, $\chi_{zxx}^{(2)}$, $\chi_{zyy}^{(2)}$, $\chi_{zzz}^{(2)}$.

$$\chi^{(2)} = \begin{bmatrix} \begin{bmatrix} 0 \\ 0 \\ \chi_{xxz}^{(2)} \end{bmatrix} & \begin{bmatrix} 0 \\ 0 \\ 0 \end{bmatrix} & \begin{bmatrix} \chi_{xxz}^{(2)} \\ 0 \\ 0 \end{bmatrix} \\ \begin{bmatrix} 0 \\ 0 \\ 0 \end{bmatrix} & \begin{bmatrix} 0 \\ 0 \\ \chi_{yyz}^{(2)} \end{bmatrix} & \begin{bmatrix} 0 \\ \chi_{yyz}^{(2)} \\ 0 \end{bmatrix} \\ \begin{bmatrix} \chi_{zxx}^{(2)} \\ 0 \\ 0 \end{bmatrix} & \begin{bmatrix} 0 \\ \chi_{zyy}^{(2)} \\ 0 \end{bmatrix} & \begin{bmatrix} 0 \\ 0 \\ \chi_{zzz}^{(2)} \end{bmatrix} \end{bmatrix} \tag{3}$$

The second harmonic intensity $I(2\omega) \propto |P(2\omega)|^2$. From Equations (1)-(3), we derive the second harmonic intensities for $I_{\varphi p}$ and $I_{\varphi s}$.

$$\begin{aligned} I_{\varphi p}(2\omega) \propto \Big(0.35cos^2(\varphi)\chi_{zzz}^{(2)} &- 0.71cos^2(\varphi)\left(0.90\chi_{xxz}^{(2)} + 0.10\chi_{yyz}^{(2)}\right) \\ &+ 0.35cos^2(\varphi)\left(0.90\chi_{zxx}^{(2)} + 0.10\chi_{zyy}^{(2)}\right) \\ &+ 0.71sin^2(\varphi)\left(0.90\chi_{zyy}^{(2)} + 0.10\chi_{zxx}^{(2)}\right) \\ &- 0.30cos(\varphi)sin(\varphi)\left(\chi_{xxz}^{(2)} - \chi_{yyz}^{(2)}\right) \\ &+ 0.30cos(\varphi)sin(\varphi)\left(\chi_{zxx}^{(2)} - \chi_{zyy}^{(2)}\right)\Big) \end{aligned} \tag{4}$$

$$I_{\varphi s}(2\omega) \propto \left(0.26cos^2(\varphi)\left(\chi_{xxz}^{(2)} - \chi_{yyz}^{(2)}\right) + 1.41cos(\varphi)sin(\varphi)\left(0.9\chi_{yyz}^{(2)} + 0.1\chi_{xxz}^{(2)}\right)\right) \tag{5}$$

We first test the BTO 9/3/10 sample which contains 19 total BTO unit cells. As shown in Supplementary Figure 3c, the red and pink curves show the SHG signal as a function of the rotating fundamental field polarization angle φ for P-polarized SHG (red) and S-polarized SHG (pink). Maximums of the measured second harmonic occur for P-polarized SHG at angles of 30º and 210º and S-polarized SHG at angles of 135º and 315º. In Supplementary Figure 3c, the raw data corresponds to the open circles and the solid lines are the theoretical fits for an *mm2* point group symmetry using equation (4) and (5). The nice fit between the experimental and theoretical data confirms the polar nature of our heterostructures as previously observed in BTO thin film [1].

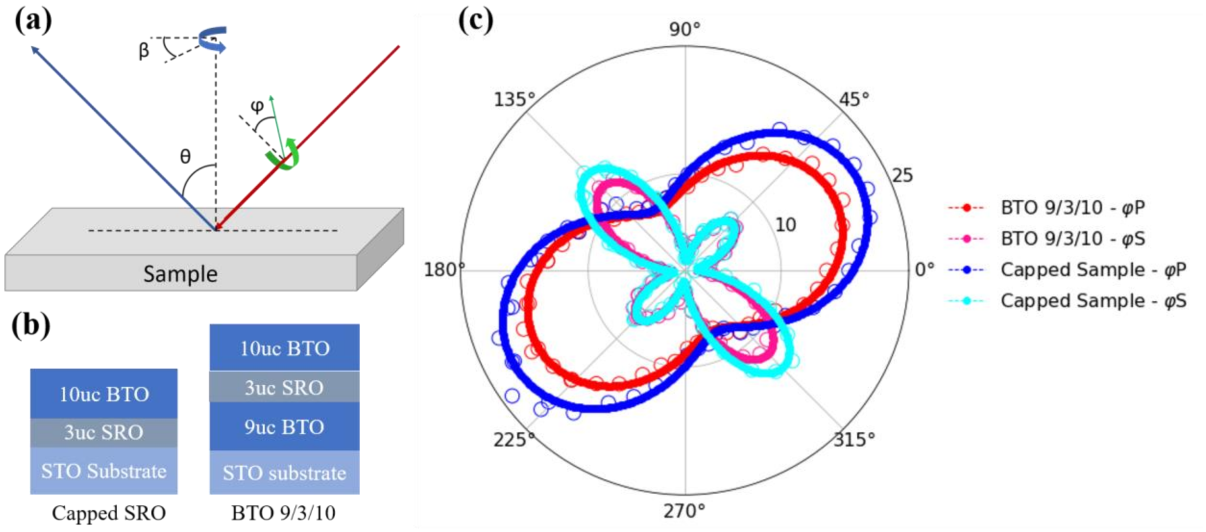

Supplementary Figure 3: Second harmonic polarimetry curves as a function of fundamental field polarization (φ) for P-polarized and S-polarized second harmonic. **a** Experimental setup. **b** Sample schematics. **c** Red (xP) and pink (xS) curves show the second harmonic intensities for BTO 9/3/10 sample, and blue (xP) and cyan (xS) curves show the second harmonic intensities for capped SRO sample.

To better understand the polar configuration in BTO 9/3/10, we performed identical measurements on a sample consisting of 3 u.c. SRO capped with 10 u.c. BTO (referenced as "capped SRO"). The data is shown as blue and cyan curves in Supplementary Figure 3. Both samples have the same symmetry indicated by the same polarization anisotropy patterns, signifying consistent BTO growth and the polar nature of both films. Noticeably, the peak intensity for the lobe maximums in the capped SRO shows a considerable increase with respect to the BTO 9/3/10, suggesting that the polar intensity is stronger in capped sample. For consistency, measurements were repeated for both samples at multiple sample positions and showed similar behaviors, ruling out inhomogeneity as the cause for the polarization anisotropy difference.

The above results reveal that the capped sample (with 10 total BTO u.c.) has stronger polar signal than BTO 9/3/10 sample (with 19 total BTO u.c.). This observation indicates that in BTO 9/3/10, the two BTO layers have opposite polar directions. If the two BTO layers in BTO 9/3/10 have parallel polar directions, one would expect the SHG intensity from BTO 9/3/10 with 19 total BTO u.c. to be stronger than the capped SRO with 10 total BTO u.c. This is not the case in Supplementary Figure 3. The experimental results in Supplementary Figure 3 are consistent with the scenario of

head-to-head polar configuration. The head-to-head configuration in BTO layers causes partial cancellation of the SHG signal, resulting in weaker SHG intensity compared to the capped sample where no such cancellation is present. We note that in BTO 9/3/10, the SHG intensity can also contain polar signal from the SRO layer, so the contribution from BTO layers is even smaller. From the SHG results, although we cannot completely rule out the existence of tail-to-tail configuration, but combined with STEM results, we conclude that on macroscopic level, the head-to-head polar direction is the dominant configuration in our heterostructure.

Supplementary Note 4: In-plane lattice parameters of the film.

The lattice parameter profile is measured from the HAADF-STEM image taken along the [100] direction, averaging 50 u.c. along the interface. As shown in Supplementary Figure 4, the in-plane lattice parameters of the BTO 9/3/10 film follow with the STO substrate, which indicate that the film grow coherently on the STO substrate under compressive strain.

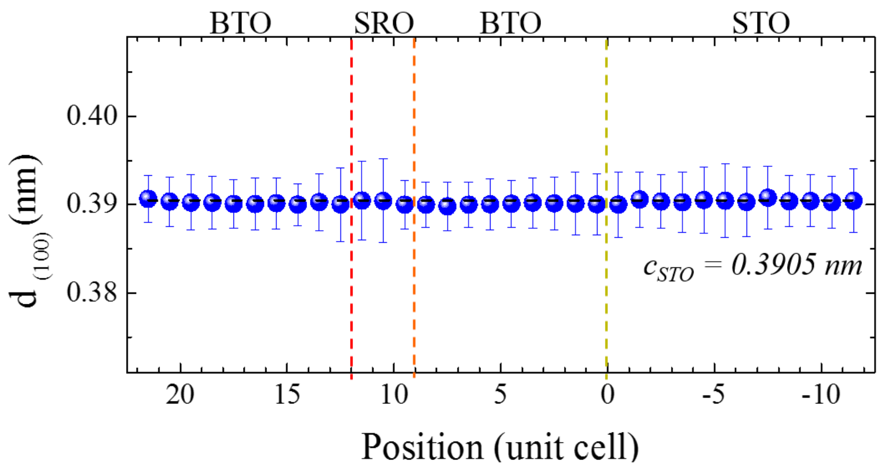

Supplementary Figure 4: In-plane lattice parameters along [100] direction, as a function of distance from the interface. The dotted black line indicates the in-plane lattice constant of bulk STO (0.3905nm). The error bar shows the standard deviations of the averaged measurements for each vertical atomic layer.

Supplementary Note 5: Defect densities and X-ray diffraction results of BTO 9/3/10 and STO 9/3/10 film.

Microscopically, we use STEM to compare BTO 9/3/10 and STO 9/3/10 sample with the same sample are of 7 μm in length and 30 nm in width. In STO 9/3/10, no dislocations are observed in the whole area. In BTO 9/3/10, only one dislocation was observed within the whole area. These results indicate that both samples are of exceptional qualities with extremely low defect densities, and the defect density of STO 9/3/10 is even lower than BTO 9/3/10.

Macroscopically, we measured the x-ray diffraction data of STO 9/3/10 as shown in Supplementary Figure 6. The reciprocal space mapping shows that the film are fully strained to the substrate. The rocking curve around the STO (002)c shows the FWHM with the value of 0.009 degree which is almost equal to BTO 9/3/10 as shown in Supplementary Figure 5. These results are consistent with our STEM data that the defect density is extremely low.

The above observations are consistent in the viewpoint of lattice mismatch. The lattice constant of bulk STO is 3.905 Å, bulk BTO is a = 3.992 Å and c = 4.036 Å, respectively. Therefore, the lattice mismatch between BTO and STO is ~ 2% in BTO 9/3/10, thus the deposition of BTO on STO substrate is under compressive strain and have stronger tendency to create defects and dislocations [3]. In comparison, the STO film grown on STO substrate are homoepitaxity so less defects and dislocations are expected. In both samples, SRO layers are grown under same condition, so the level of oxygen vacancies is expected to be the same, ruling out oxygen vacancies as the major reason to the observed phenomena. Combined with our experimental data, we conclude that both films are of good quality and low defect densities, so that the transport and magnetic properties shown in Fig. 2 and Fig. 3 represents the intrinsic behavior of our heterostructures.

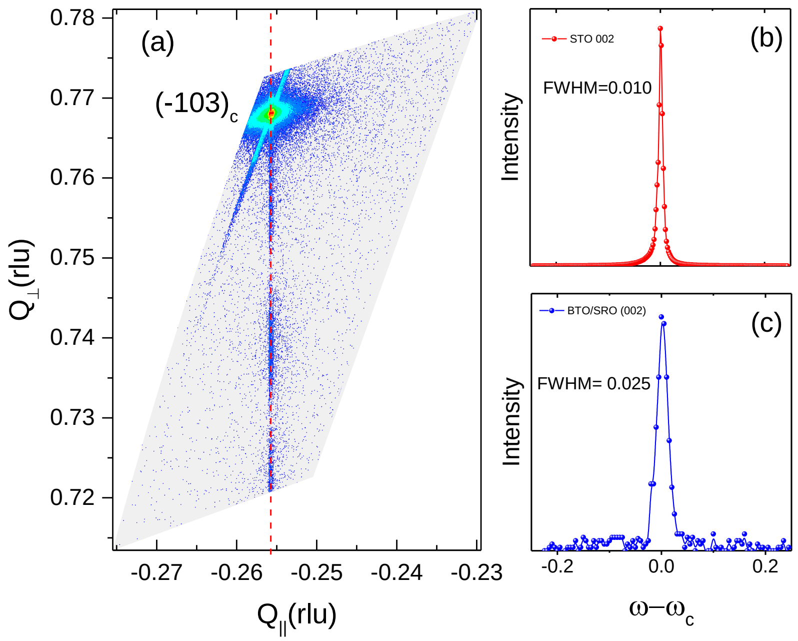

Supplementary Figure 5: a RSM around $(-103)_C$ for BTO 9/3/10 on STO (001). The thin film is fully strained to the substrate since Q_X for the thin film is the same as the substrate. **b, c** Rocking curve around thin film and substrate $(002)_C$. The FWHM of thin film and substrate show the high crystallinity of the thin film.

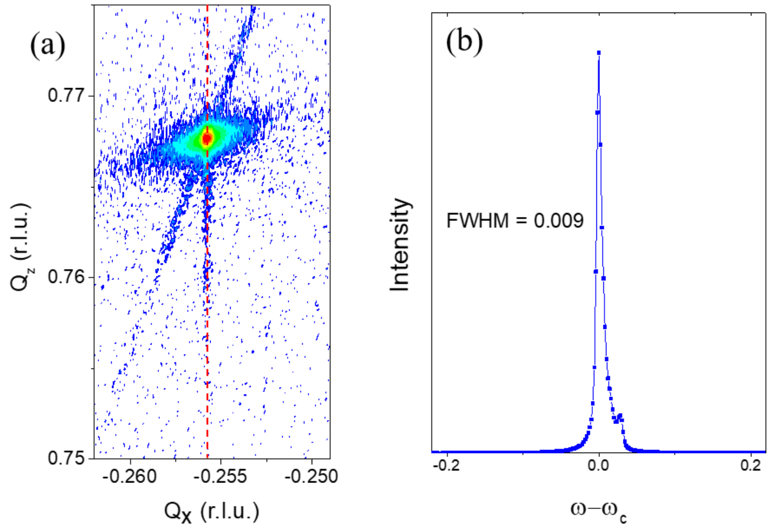

Supplementary Figure 6: a RSM around (-103)c for STO 9/3/10 on STO (001) substrate. The thin film is fully strained to the substrate since Q_X for the thin film is the same as the substrate. **b**

Rocking curve around thin film and substrate $(002)_C$. The FWHM of thin film and substrate is 0.009, which is nearly identical to the BTO 9/3/10 sample, suggesting the low defect densities in both samples.

Supplementary Note 6: Effect of capping layer

To illustrate the effect of capping layer, we fabricate a 3 u.c. SRO on STO substrate which is capped with 10 u.c. of BTO layers (refer as "capped SRO" below). As shown in Supplementary Figure 7, the capped sample shows the resistivity between 225 and 730 μΩ·cm and metal-insulator transition at 132 K. Compared with the bare 3 u.c SRO film, the capping layers can protect the SRO surface by preserving and modulating the Ru-O-Ru bonds which will improve the metallicity [4]; However from Supplementary Figure 7, the BTO capping layer alone is not enough to achieve the good metallic behavior as in BTO 9/3/10. Therefore, we argue that two bracketing BTO layers are required to achieve the conducting behavior in BTO 9/3/10.

We have also examined a BTO/STO superlattice of 40 STO and 210 BTO u.c. on STO substrate, which is grown at the same condition with the samples in manuscript. At all temperatures, the resistance is beyond the measuring limit of our instrument (> 10 Gohm). This suggests that both BTO and STO layers are of insulating nature as consistent with their intrinsic properties. Moreover, the magnetic signal of BTO/STO superlattice only contains diamagnetic background [5]. Thus the BTO/STO layers will not contribute to the ferromagnetic signal.

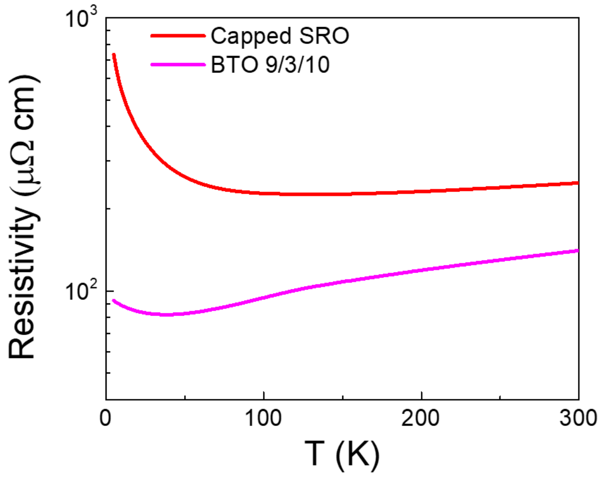

Supplementary Figure 7: resistivity vs. temperature comparison for BTO 9/3/10 and capped SRO sample (3 u.c. SRO capped with 10 u.c. BTO grown on STO substrate).

Supplementary Note 7: Multiple sample runs.

To show the causality of our data, we have run the resistivity measurements for three samples of BTO 9/3/10 under the same growth conditions. For clarity, the resistivity data are normalized to the room temperature value. As shown in the Supplementary Figure 8, all three samples show similar resistivity behavior with some variations. The residual-resistance ratio (RRR) at room temperature and 2 K (R_{300K} / R_{2K}) of three samples are 0.53, 0.65 and 0.74. In comparison, the STO 9/3/10 sample is 2.05 which is about 3-4 times larger. Moreover, the metal-to-insulator transition temperature for BTO 9/3/10 samples are 24 K, 38 K and 46K, well below 92 K for STO 9/3/10. These results suggest that our observed features are generic and reflects the intrinsic physical behavior of our heterostructures.

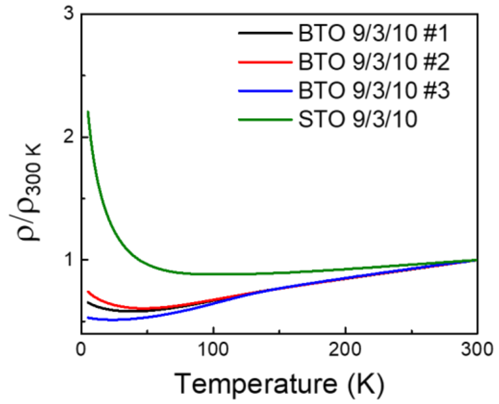

Supplementary Figure 8: Resistivity vs. Temperature for three BTO 9/3/10 samples. All three BTO 9/3/10 samples shows low resistivity compared to STO 9/3/10.

Supplementary Note 8: STEM characterization on STO 9/3/10 sample.

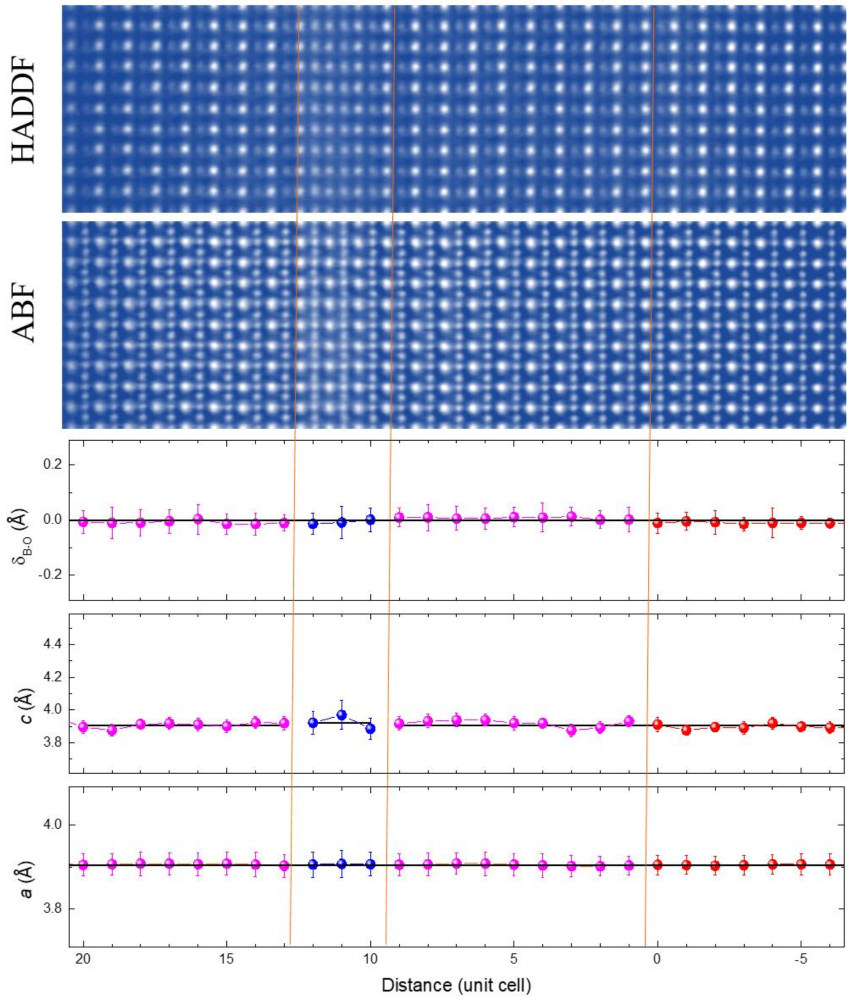

Supplementary Figure 9: STEM characterization on STO 9/3/10. Atomic-resolved HADDF and ABF images are shown. δ_{B-O} shows that no polar distortions on both STO and SRO layers are observed. The lattice constant data (a and c axis) suggests that the STO film are strain-free from the STO substrate. The error bar shows the standard deviations of the averaged measurements for each vertical atomic layer.

Supplementary Note 9: Choice of 3 u.c. SRO

We point out that the central message of this manuscript is to achieve magnetic polar metal phase, and 3 u.c. SRO is the prime layer number for the following reasons: 1) Due to the nature of interfacial coupling, the polar distortion modes are only expected to be induced when SRO is at ultrathin thickness; For example, our previous work on the BTO/LSMO/BTO superlattices shows that

polar distortions are observed when LSMO thickness is below 4 u.c [5]. 2) At ultrathin thickness region, with reduced layer number, it is well-known that both the conductivity and magnetism of SRO will suppress substantially and become completely insulating and nonmagnetic due to either dimensionality or localization effects. These effects are widely observed both in the form of bare SRO films [4,6-8] or SRO-based heterostructures [9,10]. Due to these restrictions, we argue that the 3 u.c. SRO sandwiched with BTO is the prime thickness to identify a magnetic polar metal phase in SRO layer.

Supplementary Note 10: EELS measurements on BTO 9/3/10.

The key issue on the magnetic origin is that whether the magnetism contains contribution from Ti ions in BTO layers. We have performed EELS measurements to exclude the influence of $BaTiO_3$ layers on the contribution of magnetism. We collected EELS spectra of Ti L edge across the whole film and it reveals that the oxidation state of Ti in BTO are around 4+. Supplementary Figure 10a show the EELS spectra of Ti-L edge unit cell by unit cell. The oxidation state of Ti ions can be determined from the Ti-L edge positions [11]. Using Gaussian/Lorentz fitting method, we extracted the position of e_g peaks Supplementary Figure 10b) and get the Ti oxidation states (Supplementary Figure 10c). Since the oxidation state of Ti is around 4+, the $3d^0$ orbital would not induce magnetism in $BaTiO_3$. Therefore, we conclude that the magnetism observed in our system is originated from Ru in SRO layers.

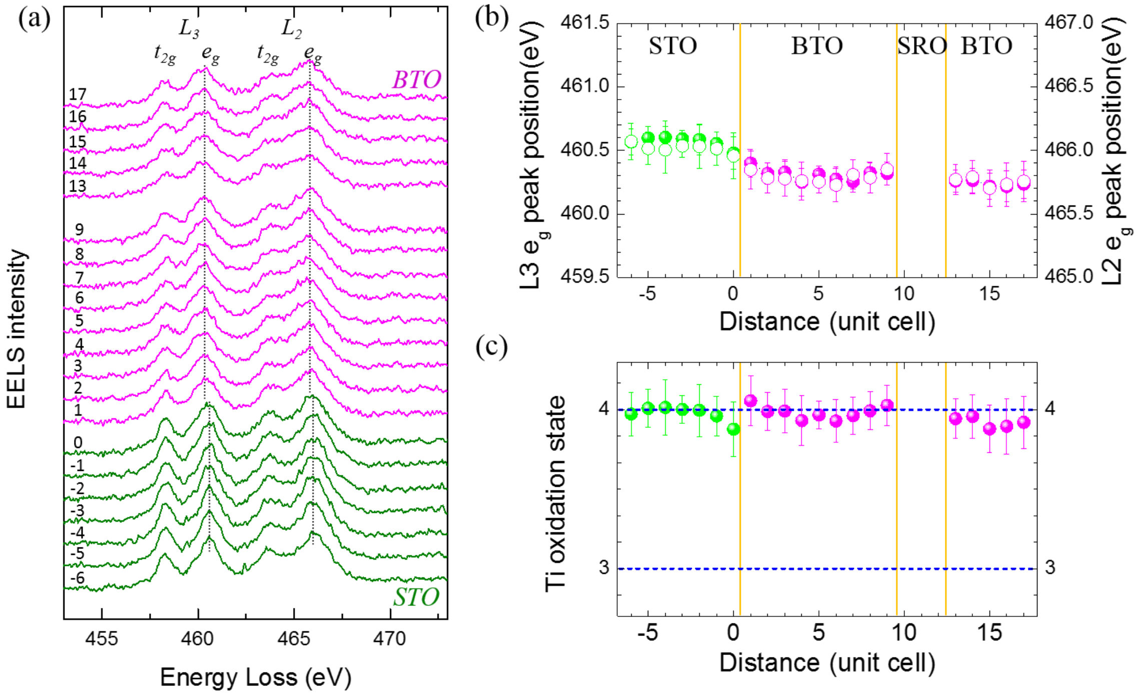

Supplementary Figure 10: a Background subtracted EELS spectra of Ti-L edge across the BTO/SRO/BTO film. The terminated TiO_2 layer of STO substrate is set as x = 0. **b** Energy position of *L3* (solid balls) and *L2* (open balls) e_g peaks extracted from the spectra using Gaussian/Lorentz fitting. **c** Oxidation state of Ti ions determined by the peak positions showing the Ti ions in BTO are all around 4+. The error bars are determined from the errors introduced in background subtraction, data fluctuation and standard deviation of spectra fitting.

Supplementary Note 11: Density functional theory.

We pursue theoretical approaches to address the observed polar displacements in SRO and their relationship to the unusual magnetic properties. In TMOs, acentric B-site displacements are common in d^0-electronic configurations[12] which are typically diamagnetic and insulating. Magnetism usually exists in antiferrodistortive (AFD) systems with d^n-electronic configurations, where acentric polar displacements are not favored since the energetic gain via $d\pi - p\pi$ metal-oxygen bonds decreases upon electron filling of the *d* orbitals[13].

Given the above, we performed first-principles density-functional-theory (DFT) and DFT+U (*d-d* static Coulomb interaction) calculations (See *Methods* and Supplementary Figure 13). In view of the high computational costs, it is not practical to model the structure used in the experiments, namely a STO/BTO_9/SRO_3/BTO_{10}/vacuum. Instead, we focus on a smaller system, BTO_6/SRO_3/BTO_7, constrain the two BTO films to have head-to-head polarization as observed in the experiments, and inquire what kind of displacements and distortions are induced in the SRO film in order to compare with the experimental data (Supplementary Figure 11a). We adopted a supercell with the atomic structure shown in Supplementary Figure 11b. Periodic boundary conditions turn it into a BTO_{13}/SRO_3 superlattice. In order to build in the head-to-head polarization, we froze the first and last atomic layers with zero displacements in Supplementary Figure 11b, which is equivalent to freezing with zero displacements atomic layers #6 and 7 in the BTO_{13} layers of the BTO_{13}/SRO_3 superlattice (see Supplementary Figure 14 for the illustration of periodic boundary conditions of the adopted supercell). As already stated, our objective is to probe how the SRO film behaves when sandwiched between the head-to-head configuration and compare with the experimental data. We note that a true 2D model where the periodicity is allowed in-plane but terminated along the growth direction through a vacuum region could be more efficient for our system. However, we found that using this 2D model, the end BTO layers also need to be fixed during relaxation to ensure opposite polar displacements in the BTO blocks, similar to the 3D model we have performed. A similar approach has also been taken in a recent paper[14].

We have considered three initial structure configurations by inducing different possible modes (Q) in the $SrRuO_3$ block guided by the symmetry to obtain the optimized low symmetry geometry. In Supplementary Figure 11b, structure (i) shows an initial geometry where a polar mode (similar to BTO) is frozen in the SRO block, defined as $\langle P+|Q_{Pol}|P-\rangle$. Structure (ii) shows an initial geometry where we freeze in the bulk *Pnma* AFD a-a-c+ distortion in the SRO block defined as $\langle P+|Q_{AFD}\,|P-\rangle$. This AFD mode is further described by three symmetrized basis modes of the cubic *Pm-3m*: Q_{Rot}, in-plane rotation of the BO_6 octahedra about [001]; Q_{Tilt}, tilt of BO_6 octahedra about [110]; and antiferroelectric A-site displacements. Finally, the third initial structure is a combination of (i) and (ii), i.e., Q_{Pol} + QAFD. We have optimized each of the three initial structures using various U values on Ru-4*d* ranging from 1.0 eV – 5.5 eV to study the energetics (Supplementary Figure 15). The motivation of the calculations is to study the relative stability of the possible configurations. Our results show that the bulk 'U' value of 0.6 eV is unable to explain the experimental observations in the present case[15].

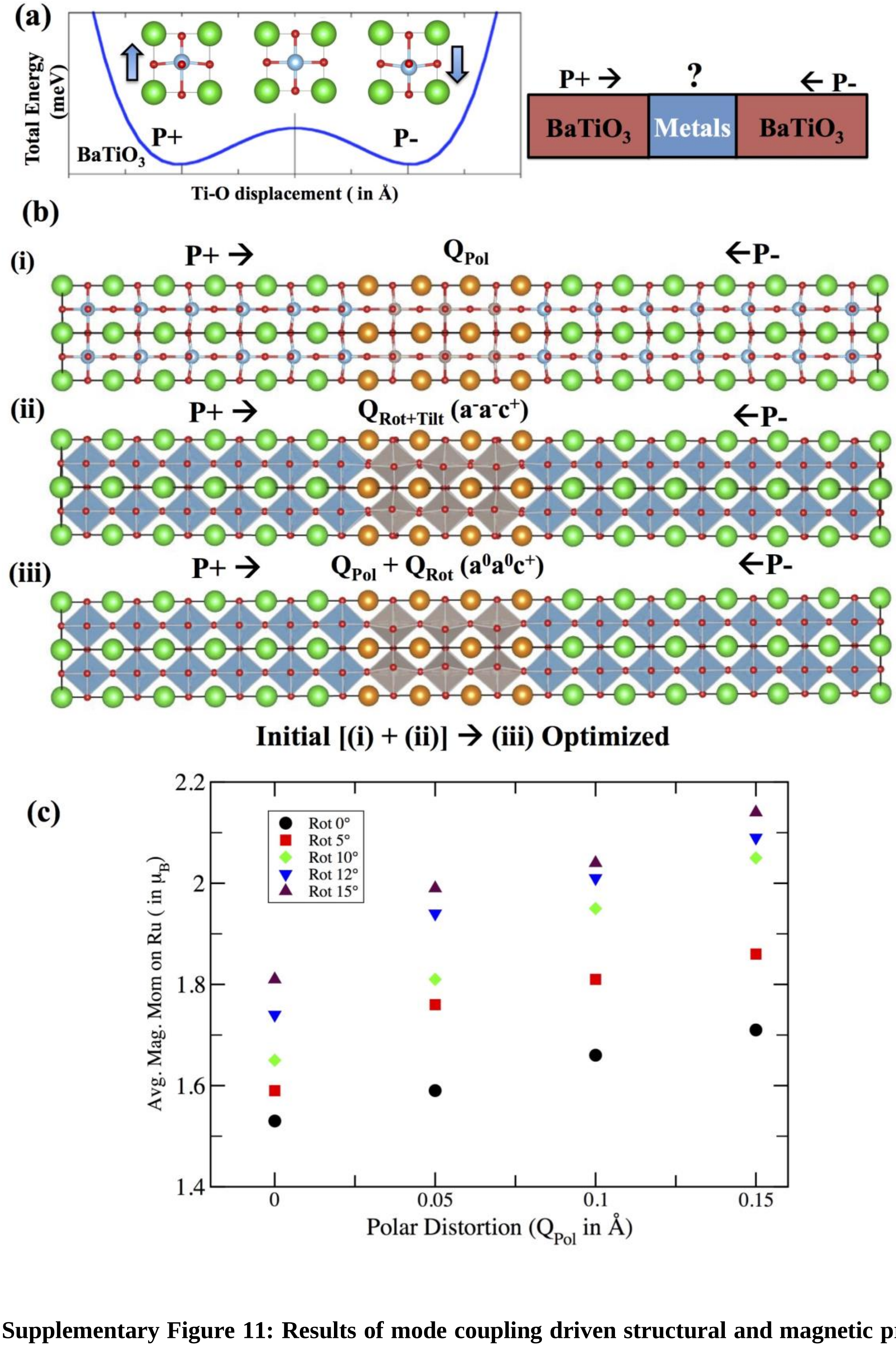

Supplementary Figure 11: Results of mode coupling driven structural and magnetic properties from DFT calculations. a Illustration of a ferroelectric (FE) mode in BTO. A metal block is

sandwiched between two equivalent minima P+ and P- of FE $BaTiO_3$. **b** Configuration (i) shows initial geometry where a ferroelectric-like (FEL) mode is considered in the SRO block sandwiched between P+ and P- <P+/Q_{FEL}/P->. The middle atomic layer in the SRO block kept fixed and FEL mode is 'frozen in' at the interface SRO layer. In configuration (ii), bulk *Pnma* distortion ($a^-a^-c^+$) that has both rotation and tilt ($Q_{Rot+Tilt}$) has been 'frozen in' in the SRO block. Finally the optimized structure is shown in (iii) for U= 3.5 eV (on Ru), where U is static *d-d* Coulomb interaction [the initial configuration is constructed from (i) + (ii)]. After optimization, structural mode analysis of the final geometry reveals the suppression of Tilt ($a^-a^-c^0$) and emergence of FEL, leading to Q_{FEL} + Q_{Rot} ($a^0a^0c^+$) distortion. **c** Variation of the average magnetic moment on Ru as a function of Q_{Pol} for different Q_{Rot} angles for U= 3.5 eV (on Ru). We have attached the CIF files of the three structural models in **b** as supplementary files.

After optimization of atomic positions, we obtained the respective structures where individual polar and AFD distortions are retained for Supplementary Figure 11 (i) and (ii), respectively. In the case of the initial starting configuration Q_{Pol} + Q_{AFD}, we found that for U $\geq$ 3.5 eV the optimized structure resulted in Q_{Pol} + Q_{Rot} as shown in Supplementary Figure 11 (iii): The polar mode emerges with complete suppression of tilt distortions ($Q_{Tilt} \sim 0.1°$) while the rotation mode (Q_{Rot}) persists which is consistent with experimental observation. Hence in the subsequent section we have discussed the results for U= 3.5 eV. The resultant structure is shown as the third configuration in Supplementary Figure 11 (iii) as <P+/Q_{Pol}+Q_{Rot} /P->. Structural analysis reveals that in the middle SRO layer, the O2-Ru2-O3 bond length is symmetric (2.20 Å) and there is no off-centering in the Ru atom. However, asymmetry in the O1-Ru2-O2 (or O3-Ru3-O4) bond length is found at the interface SRO layers (2.00 Å and 2.15 Å, respectively), which establishes considerable off-centering of Ru from the center of symmetry with amplitude about 0.15 Å (Supplementary Figure 16). These results demonstrate that polar displacements accompanied by in-plane rotations and the complete suppression of tilt distortions are favored in SRO layers, in good agreement with the STEM data.

To unveil the role of structure (modes) – property (magnetization) relationship, we calculated the magnetic moment in the ferromagnetic state. We found that the magnetic moment is 2.1 μ_B/Ru for <P+/Q_{Pol}+Q_{Rot} /P-> and in excellent agreement with the experimental data. We note, however, that the calculated value of the magnetic moment is not a true prediction because of the many constraints we imposed on our model in accord with experimental data and the value of U was adjusted. Further, we have investigated the correlation between the polar distortions with the average magnetic moment on Ru. As shown in Supplementary Figure 11c, both Q_{Rot} and Q_{Pol} is found to be responsible for driving

the enhancement in the magnetic moment. At fixed rotation angles, the enhanced polar distortions increase the magnetic moment of SRO. These features suggest that the polar distortions are responsible for the emergence of high magnetic moment in SRO.

Supplementary Figure 12 shows the total and partial density of states (DOS) of <P+/Q_{Pol}+Q_{Rot} /P-> for U= 3.5 eV. The electronic structure calculations reveal a finite DOS at the Fermi level (E_F) as shown in Supplementary Figure 12a as a signature of metallicity. The finite DOS at E_F originates from the Ru-3*d*-O-2*p* hybridized orbitals at the BTO-SRO interface. Partial DOS analysis shows that the finite DOS at E_F is due to Ru-3*d* states while the Ti-3*d* states are away from E_F. Analysis of the layer-resolved partial DOS of Ru-3*d* for interfacial Ru and middle Ru layer respectively reveals that for both cases, the up-spin t_{2g} state is completely occupied, up-spin e_g state is partially occupied, while both down-spin t_{2g} and e_g states are completely empty i.e. the $t_{2g}(3\uparrow)e_g(1\uparrow)$ electronic configuration, which should give rise to a local magnetic moment close to 4 μ_B on each Ru. Due to strong hybridization between Ru-3*d* and O-2*p*, the average local magnetic moment on Ru is reduced. Still, the average magnetic moment is found to be > 2 μ_B for U=3.5 eV, consistent with magnetization measurements. The results in Supplementary Figure 11 and12 suggest the role of electronically mediated spin-lattice coupling in the present system.

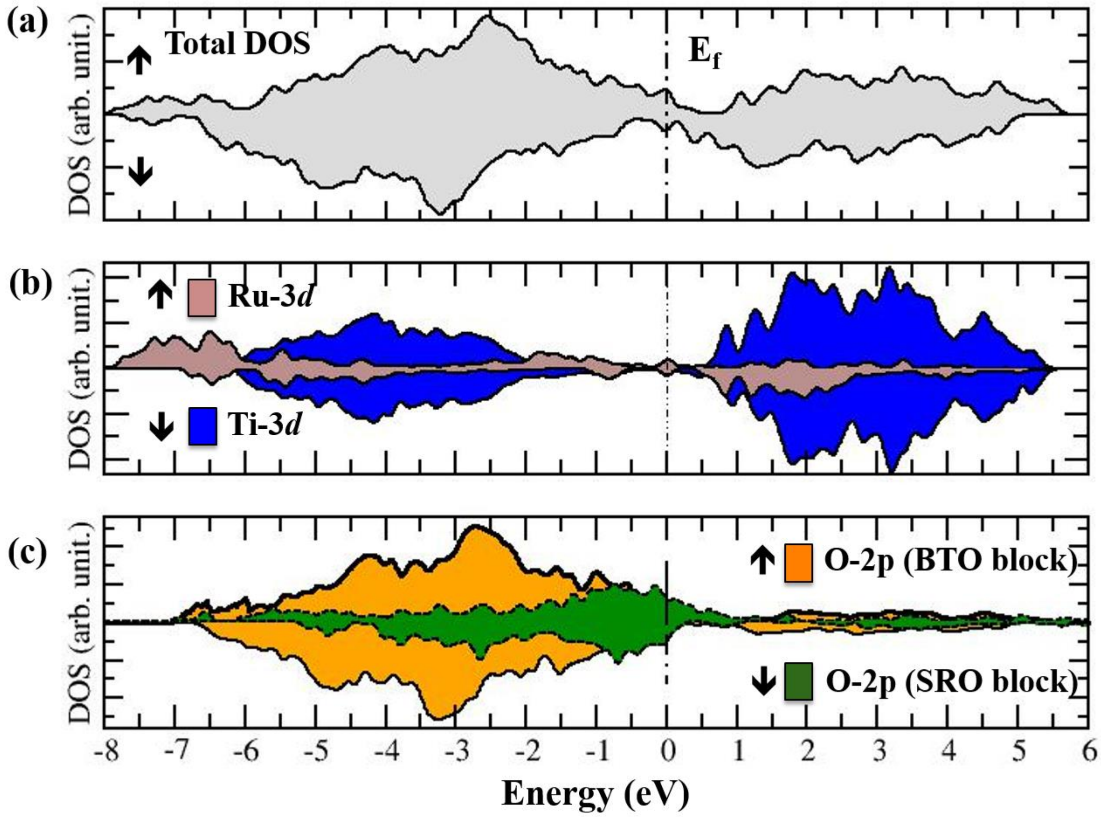

Supplementary Figure 12: Density of States from first-principles DFT calculations. a, **b** and **c** represent total, transition metal-3*d* (Ru and Ti) and O-2*p* projected DOS respectively at U = 3.5 eV.

Supplementary Note 12: Supercell structure.

We considered only the head-to-head <P+/SrRuO3/P-> configuration and sought to identify the structure forced on the SRO layer by the experimentally observed head-to-head BTO layers. In order to ensure a periodically repeated supercell, we use zero displacements in the last atomic layer of the BTO films shown in the schematic of Supplementary Figure 11b. In other words, as shown in Supplementary Figure 14 below, if we look at the continuous 13-atomic-layer BTO that results from imposing periodic boundary conditions, layers #6 and #7 are frozen without displacements, leaving us essentially with a <5+/SRO/6-> structure.

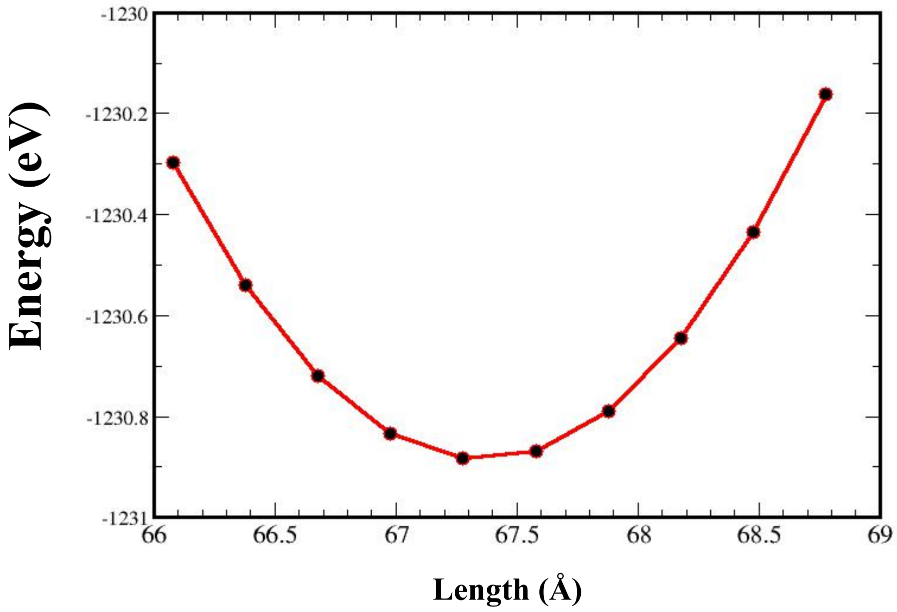

Supplementary Figure 13: Energy vs. length of simulation cell along c axis of <P+/$SrRuO_3$ (Cubic) /P->

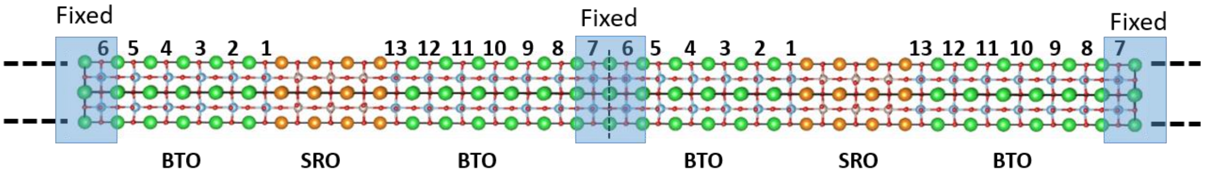

Supplementary Figure 14: Illustration of periodic boundary conditions of the adopted supercell. In the view of continuous 13 BTO layers, #6 & #7 are frozen without displacements. Next, we impose negative atomic displacements in layers 1-5 and positive atomic displacements in layers 8-13 to model the experimentally observed P-/SRO/P+ structure. Relaxation of the SRO film then produces polar displacements in accord with the experimental observations, confirming that SRO in this structure is indeed a polar metal.

Supplementary Note 13: Variation of 'U' on the distortion and magnetic properties

As shown in Supplementary Figure 15, suppression of Q_{Tilt}, emergence of Q_{Pol} distortion and enhancement of the magnetization has been observed when U ≥ 3.5 eV.

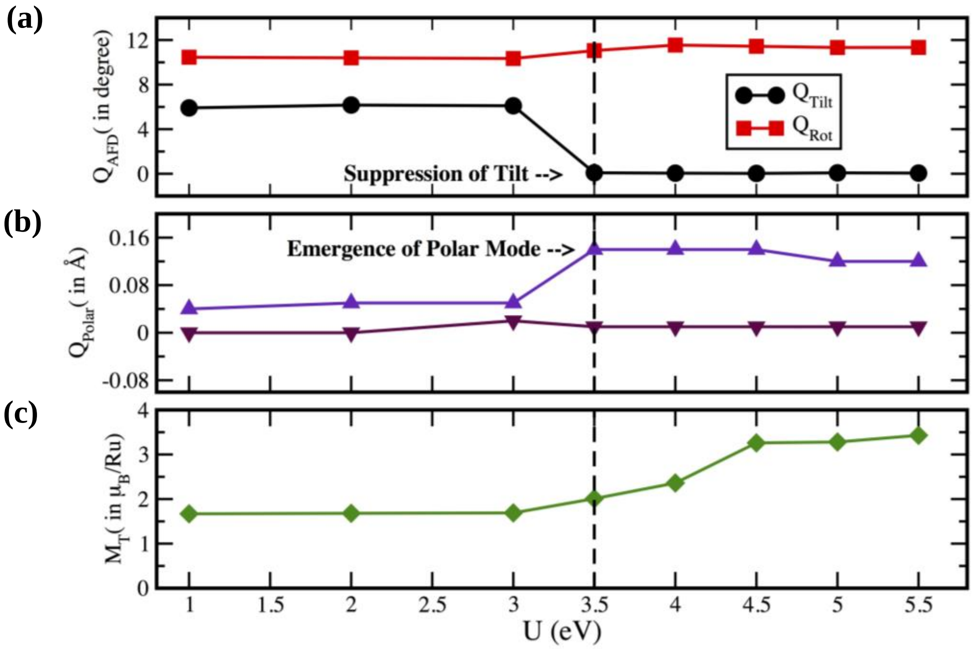

Supplementary Figure 15: Variation of **a** Q_{AFD}, **b** Q_{Polar} and **c** magnetic moment on Ru with respect to DFT+U (*d-d* static Coulomb interaction)

Supplementary Note 14: Estimation of the induced polar distortion in SRO block, O-Ru-O bond length variation

In SRO block, structural analysis reveals that in the middle SRO layer, the O2-Ru2-O3 bond length is symmetric (2.20 Å) and there is no off-centering in the Ru atom. However, asymmetry in the O1-Ru2-O2 (or O3-Ru3-O4) bond length is found at the interface SRO layers (2.00 Å and 2.15 Å, respectively), which establishes considerable off-centering of Ru from the center of symmetry with amplitude about 0.15 Å. The results are shown in Supplementary Figure 16.

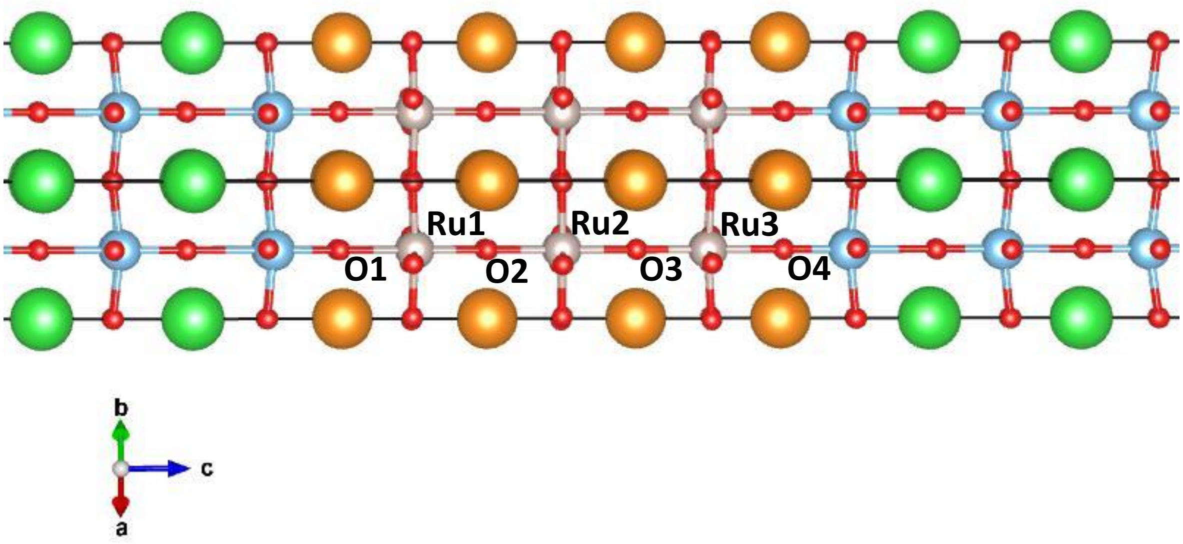

Supplementary Figure 16: Optimized <P+/SRO (Rot + Polar) /P-> configuration

Supplementary References:

[1] Cao, Y. et al. Artificial two-dimensional polar metal at room temperature. *Nat. Commun.* **9,** 1547 (2018).

[2] Shen, YR, The principles of nonlinear optics. Wiley-Interscience, New York (1984).

[3] Sun, H. P. et al. Evolution of dislocation arrays in epitaxial $BaTiO_3$ thin films grown on (100) $SrTiO_3$. *Appl. Phys. Lett.* **84**, 3298 (2004).

[4] Chang, Y.J. et al. Fundamental thickness limit of itinerant ferromagnetic $SrRuO_3$ thin films. *Phys. Rev. Lett.* **103**, 057201 (2009).

[5] Guo, H. et al. Interface-induced multiferroism by design in complex oxide superlattices. *Proc. Natl. Acad. Sci. (PNAS)* **114**, E5062 (2017).

[6] Ishigami, K. et al. Thickness-dependent magnetic properties and strain-induced orbital magnetic momentin $SrRuO_3$ thin films. *Phys. Rev. B* **92**, 064402 (2015).

[7] Jing, X. et al. Critical thickness for itinerant ferromagnetism in ultrathin films of $SrRuO_3$. *Phys. Rev. B* **79**, 140407(R) (2009).

[8] Shen, X. et al. Thickness-dependent metal-insulator transition in epitaxial SrRuO3 ultrathin films. *J. Appl. Phys.* **117**, 015307 (2015).

[9] Liu, Z.Q. et al. Tailoring the electronic properties of $SrRuO_3$ films in $SrRuO_3$/$LaAlO_3$ superlattices. *Appl. Phys. Lett.* **101**, 223105 (2012).

[10] Bern, F. et al. Structural, magnetic and electrical properties of $SrRuO_3$ films and $SrRuO_3$/$SrTiO_3$ superlattices. *J. Phys.: Condens. Matter* **25**, 496003 (2013).

[11] Shao, Y., Maunders, C., Rossouw, D., Kolodiazhnyi, T., & Botton, G. A., Quantification of the Ti oxidation state in $BaTi_{1-x}Nb_xO_3$ compounds. *Ultramicroscopy* **110**, 1014 (2010).

[12] Puggioni, D. & Rondinelli, J. M. Designing a robustly metallic noncenstrosymmetric ruthenate oxide with large thermopower anisotropy. *Nat. Commun.* **5,** 3432 (2014).

[13] Kunz, M. & Brown, I. D. Out-of-Center Distortions around Octahedrally Coordinated d^0 Transition Metals. *J. Solid State Chem.* **115,** 395–406 (1995).

[14] Reddy, I. R., Oppeneer, P. M. & Tarafder, K. Route to achieving giant magnetoelectric coupling in $BaTiO_3$/Sr_2CoO_3F perovskite heterostructures. *Phys. Rev. B* **98,** 140401 (2018).

[15] Rondinelli, J. M., Caffrey, N. M., Sanvito, S. & Spaldin, N. A. Electronic properties of bulk and thin film $SrRuO_3$: Search for the metal-insulator transition. *Phys. Rev. B* **78,** 155107 (2008).
