# Supplementary material for: Interface-induced magnetic polar metal phase in complex oxides
Source: Nat Commun. 2019 Nov 20;10:5248. doi: 10.1038/s41467-019-13270-7 (PMC6868157; doi:10.1038/s41467-019-13270-7)
Supplement: Supplementary file 2 — Description of Additional Supplementary Files [file 41467_2019_13270_MOESM2_ESM.pdf]

Title: Supplementary Data 1
Description: Initial_Polar

Title: Supplementary Data 2
Description: Initial_Rotation+Tilt

Title: Supplementary Data 3
Description: Final_Polar+Rotation
